# Antibacterial Activity of Synthetic Precursors of Podophyllotoxin

**Published:** 2007-06

**Authors:** N. Nanjundaswamy, S. Satishi, K. M. Lokanatha Rai, S. Shashikanth, K. A. Raveesha

**Affiliations:** 1 *Department of Studies in Chemistry, University of Mysore, Manasagangotri, Mysore, India;*; 2 *Department of Studies in Microbiology, University of Mysore, Manasagangotri, Mysore, India*

**Keywords:** podophyllotoxin, antibacterial activity, human pathognic

## Abstract

Precursors of podophyllotoxin were synthesized and screened for their antibacterial activity. The results proved that ethyl-2-(3′-methyl-4′-methoxybenzoyl)-3-(4″ methoxyphenol)-cyclopropane-1-carboxylic acid and Ethyl-2-(3′-methyl-4′-methoxybenzyol-3-1 3″, 4″-dimethoxyphenyl)-cyclopropane-carboxylic acid have significant antibacterial activity against *Citrobacter* sp., *Escherchia coli, Klebsiella pneumoniae, Pseudomonas aeruginosa, Salmonella typhi, Shigella sonnei* and *Streptococcus faecalis*. The activity is lower than Ciprofloxacin and equal to Gentamicin and more than Penicillin and Streptomycin.

## INTRODUCTION

**Figure 1:**
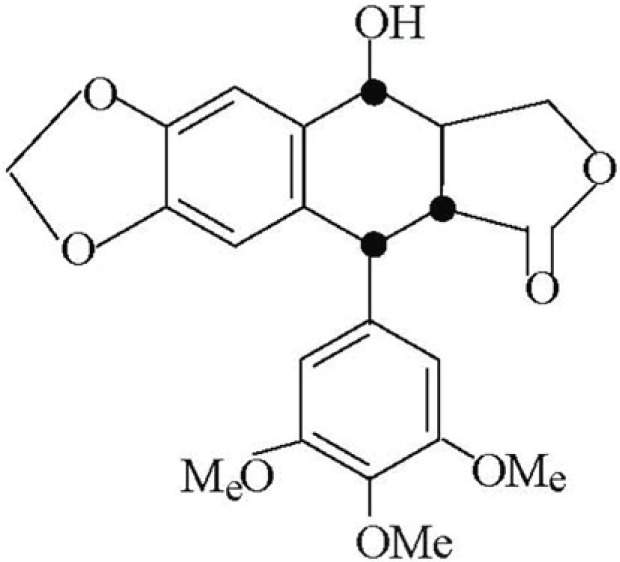
Chemical structure of podophyllotoxin.

**Figure 2:**
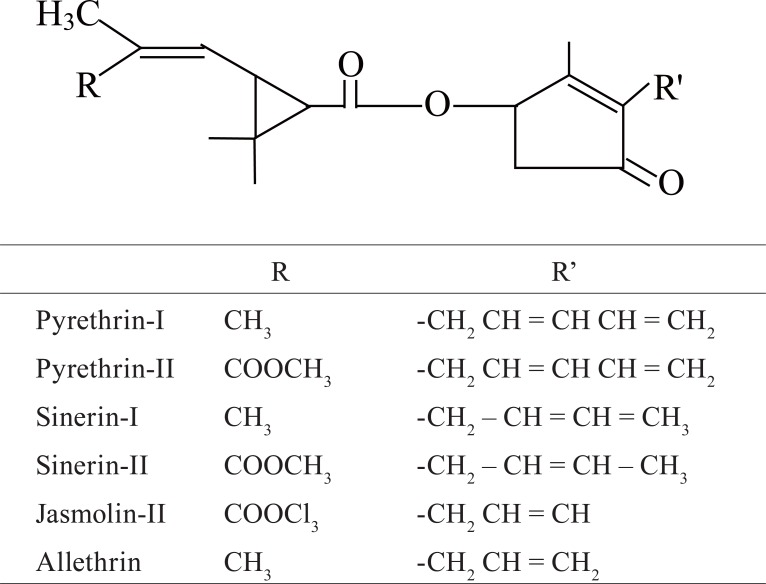
Chemical structures of pythroid derivatives tested in the current study.

Podophyllotoxin (Figure 1) and several of its analogs are being used as cytotoxic spindle poisons and antitumor agents at clinical levels (1, 2). Recently Lee *et al*. (3) discovered that some modified derivatives of podophyllotoxin possess an anti HIV property. Most of these compounds contain a transfused highly stained γ-lactone system (4), a feature that correlates with the smooth Isomerisation of podophyllotoxin to its thermodynamically stable epimer, picropodophyllin. The biological activity of *cis* analogous are very much low than that of the *trans* isomer (5). Literature survey on the structure and activity relationship among the podophyllin derivatives revealed efforts have not been made towards the study of the other biological activities in general and antibacterial activity in particular. Literature studies (6) also reveal that prythroid derivatives (Figure 2) possessing cyclopropam ring system show strong insecticidal property.

**Figure 3:**
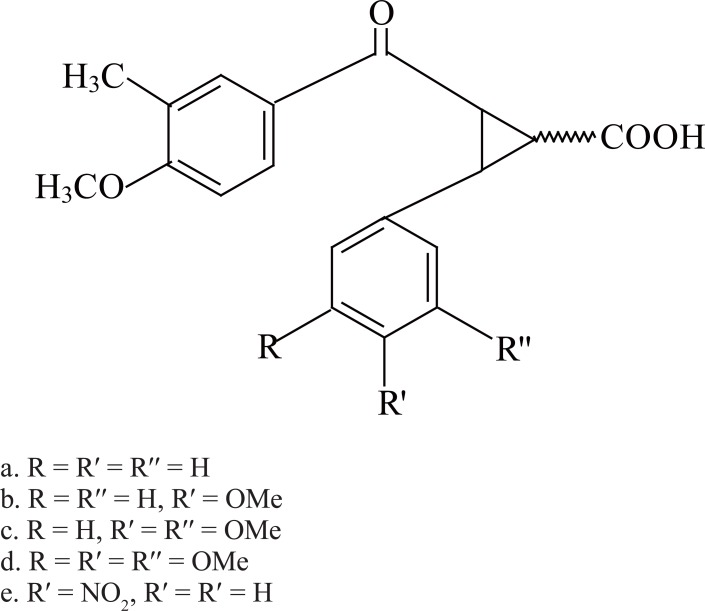
Molecules based on the cyclopropane ring system used in the current study as the synthetic precursor for synthesis of podophyllotoxin derivatives.

These results prompted us to synthesize the molecules from cyclopropane ring system, which can serve as a synthetic precursor (Figure 3) for the synthesis of podophyllotoxin derivatives and evaluated their antibactericidal potential against some important human pathogenic bacteria.

## MATERIALS AND METHODS

The following compounds (3a-, 3b-, 3c-, 3d- and 3e) were synthesized by cyclopropanation of chalcones using the literature method (7).

3a. 2-(3′-methyl-4′-methoxybenzoyl)-3-phenyl-cyclopropane-1-carboxylic acid.

3b. 2-(3′-methyl-4′-methoxybenzoyl)-3-(4′′-methoxyphenyl)-cyclopropane-1-carboxylic acid.

3c. 2-(3′-methyl 1-4′-methoxybenzoyl)-3-(3′′, 4′′-dimethoxyphenyl)–cyclopropane-1-carboxylic acid.

3d. 2-(3′-methyl-4′-methoxybenzoyl)-3-(3′′, 4′′, 5′′-trimethoxyphenyl)-cyclopropane-1-carboxylic acid.

3e. Ethyl-2-(3′-methyl-4′-methoxybenzoyl)-3-(5′′-nitrophenyl)-cyclopropane-1-carboxylic acid.

### Bacterial cultures

*Citrobacter* sp., *Escherchia coli* (MTCC443), *Klebsiella pneumoniae* (MTCC109), *Pseudomonas aeruginosa* (MTCC1688), *Salmonella typhi* (MTCC733), *Shigella sonnei* (MTCC2957) and *Streptococcus faecalis* (MTCC459) obtained from Microbiology laboratory. Government Medical College, Mysore, India served as test organisms for antibacterial activity assay.

### Antibacterial assay

**Preparation of nutrient broth.** Nutrient broth was prepared by dissolving peptic digest of animal tissue (0.5%), yeast extract (0.15%) beef extract (0.15%) and sodium chloride (0.50%) in distilled water (100 ml). The pH of solution was adjusted to 7.4 by adding sodium hydroxide solution (4%) and the resulting solution was autoclaved for 20 minutes at 15 psi.

**Preparation of sub cultures (Inoculums).** One day prior to the experiment, the cultures of *Citrobacter* sp., *Escherichia coli, Klebsiela* sp., *Pseudomonas aeruginosa, Salmonella typhi, Shigella sonnei, and Streptococcus faecalis* was inoculated to nutrient broth and incubated over night at 37°C.

**Preparation of Nutrient agar medium.** Nutrient agar medium was prepared by dissolving peptic digest of animal tissue (0.5%), yeast extract (0.15%), beef meet extract (0.15%), sodium chloride (0.5%) in distilled water (100 ml). The pH of the solution was adjusted to 7.2 by adding 4% aqueous sodium hydroxide solution and agar (1.5%) was added and autoclaved for 20 minutes at 15 psi.

**Preparation of test sample solution.** Each test sample (5 mg) was dissolved in ethyl alcohol (5 ml) and 0.01 ml of this solution (10 µg) was used for testing.

### Method of testing

Antibacterial activity was determined by cup diffusion method on nutrient agar medium. The sterile medium (20 ml) was poured into 9 cm petriplates. The medium was allowed to cool in a sterile condition and plates were then inoculated with 1 × 10^5^ cfu cultures of tested bacteria. The concentration of bacterial cells in the suspension was adjusted to minimum of 1 × 10^5^ cfu/ml in nutrient broth solution. By punching the agar container with sterile cork borer and scooping out the punched part agar cup of 5 mm diameter were made.

Test solution (0.01 ml) containing 10 µg of the test compounds were placed in each cup, ethyl alcohol (10 µl) served as control. The plates were left to stay for an hour in order to facilitate the diffusion of the drug solution. Then the plates were incubated at 37°C for 24 hours. The zones of inhibition if any against the test bacteria were measured in mm. Ciprofloxacin, Gentamicin, Penicillin G and Streptomycin were used for comparison.

### Minimal Inhibitory Concentration (MIC)

MIC was determined by broth dilution methods. For broth dilution tests, 0.1 ml of standardized suspension of test bacteria (10^5^ CFU/ml) was added to each tube containing different concentrations of the podophyllotoxin derivatives and antibiotics (05-30 μl/ml) incubated for 24hours at 37°C. The lowest concentration of the tube that did not show any visible growth by macroscopic evaluation was considered as the MIC.

## RESULTS

The antibacterial activity of all the synthetic derivatives and the antibiotics against all the test bacteria presented in Table 1.

**Table 1:** Antibacterial activity of podophyllotoxin derivatives and antibiotics

Sl. No.	Bacteria	3a	3b	3c	3d	3e	C	G	P	S

1	*Citrobacter* sp.	0.00	11.66 ± 0.24	11.00 ± 0.36	0.00	0.00	19.62 ± 0.18	12.68 ± 0.22	0.00	0.00
2	Escherichia coli	0.00	10.33 ± 0.23	10.66 ± 0.24	0.00	0.00	10.00 ± 0.25	10.25 ± 0.25	0.00	13.50 ± 0.36
3	Klebsiella pneumoniae	0.00	11.66 ± 0.24	12.16 ± 0.51	0.00	0.00	20.25 ± 0.16	11.75 ± 0.25	0.00	09.25 ± 0.16
4	Pseudomonas aeruginosa	0.00	11.00 ± 0.73	11.33 ± 0.24	0.00	0.00	34.25 ± 0.16	12.63 ± 0.18	0.00	0.00
5	Salmonella typhi	0.00	11.66 ± 0.24	12.00 ± 0.00	0.00	0.00	20.25 ± 0.16	17.75 ± 0.25	0.00	0.00
6	Shigella sonnei	0.00	13.33 ± 0.24	11.66 ± 0.24	0.00	0.00	21.75 ± 0.16	15.25 ± 0.25	0.00	0.00
7	Streptococcus fecalis	0.00	09.83 ± 0.15	11.50 ± 0.27	0.00	0.00	10.00 ± 0.25	12.50 ± 0.18	0.00	0.00

C, Ciprofloxacin; G, Gentamicin; P, Penicillin; S, Streptomycin, *p*<0.05. Values given are mean standard error of 3 replicates.

The three derivatives namely 3a, 3d, 3e and antibiotic penicillin G did not reveal any antibacterial activity, whereas the other two derivates namely 3b and 3c recorded significant activity against all the test bacteria. Streptomycin was active only against *E. coli* and *Klebsiella pneumoniae.* Ciprofloxacin and Gentamicin were active against all the test bacteria.

Antibacterial activity was maximum against *Shigella sonnei* (13.33 mm) and was minimum against *Streptococcus faecalis* (9.83 mm) in 3b derivative. In case of 3c derivative the antibacterial activity was more against *Klebsiella pneumoniae* (12.16 mm) and less against *E. coli* (10.66 mm). Antibiotic Streptomycin recorded higher activity in comparison with the derivatives 3b and 3c against *E. coli. Salmonella typhi* (17.75 mm) was highly susceptible to Gentamicin followed by *Shigella sonnei* (15.25 mm) and *Citrobacter* sp. (12.68 mm). The zone of inhibition was higest in case of *Pseduomonas aeruginosa* (34.25 mm) against Ciprofloxacin followed by *Shigella sonnei* (21.75 mm) *Salmonella typhi* (20.25 mm) and *Klebsiella pneumoniae* (20.25 mm). Lowest inhibitory activity was also observed against *E. coli* and *Streptococcus fecalis*.

Minimal inhibitory concentration of all the five derivatives of Podophyllotoxin and antibiotics tested against the test bacteria are present in Table 2. It is observed that among the antibiotic tested Ciprofloxacin was highly active with low MIC of 10 µl against *Citrobacter* sp., *Klebsiella pneumoniae, Shigella sonnei* and *Salmonella typhi*. Gentamicin also recorded low MIC value of 10 µl against *Salmonella typhi.* Streptomycin active only against *E. coli* and *Klebsiella pneumoniae* where as penicillin was not active against all the test bacteria. Among the synthetic derivatives 3b and 3c were active against all the test bacteria with MIC ranging between 15-25 µl where as 3a, 3d and 3e did not show any activity against all the test bacteria.

**Table 2:** Minimal inhibitory concentration of podophyllotoxin derivatives and antibiotics

Sl. No.	Bacteria	3a	3b	3c	3d	3e	C	G	P	S

1	*Citrobacter* sp.	-	20	20	-	-	10	20	-	-
2	Escherichia coli	-	20	20	-	-	20	20	-	15
3	Klebsiella pneumoniae	-	20	15	-	-	10	20	-	25
4	Pseudomonas aeruginosa	-	20	20	-	-	05	20	-	-
5	Salmonella typhi	-	20	20	-	-	10	10	-	-
6	Shigella sonnei	-	15	20	-	-	10	15	-	-
7	Streptococcus fecalis	-	25	20	-	-	20	20	-	-

C, Ciprofloxacin; G, Gentamicin; P, Penicillin; S, Streptomycin. Values given are mean of 3 replicates.

The MIC values of Ethyl-2-(3′-methyl-4′-methoxybenzoyl)-3-(4′′ methoxyphenyl)-cyclopropane-1-carboxylic acid and Ethyl-2-(3′-methyl-4′-methoxybenzyol)-3-(3′′, 4′′-dimethoxyphenyl)-cyclopropane-carboxylic acid was almost equal to that of Gentamicin and slightly higher than that of Ciprofloxacin against the test bacteria. Further it is observe that 3b and 3c derivatives were better antibacterialy active than penicillin and streptomycin.

## DISCUSSION

Continuous and frequent use of antibiotics to mange human disease has caused by bacteria to acquire resistance against the commonly used therapeutic agents (9). Consequently the problem of microbial resistance is growing and the need to search for alternative newer molecules is increasing. Hence there is a continuous need to evaluate for newer molecules that are antibacterialy active. Hence in the present study all the five new synthesized derivatives (3a-3e) of podophyllotoxin and their analogues are already in use as antitumor agent (1, 2), and also known to posses anti HIV property (3). None of the evaluation had demonstrated the antibacterial potential of the five synthesized derivatives tested in the present investigation. The present investigation has demonstrated clearly the antibacterial potential of 3b and 3c derivatives for the first time also has demonstrated the inefficacy of 3a, 3d and 3e.

The comparative evaluation of the synthetic derivatives with that of the commonly used antibiotics reveal that Ethyl-2-(3′-methyl-4′-methoxybenzoyl)-3-(4′′ methoxyphenyl)-cyclopropane-1-carboxylic acid and Ethyl-2-(3′-methyl-4′-methoxybenzyol)-3-(3′′, 4′′-dimethoxyphenyl)-cyclopropane-carboxylic acid were active against many penicillin and streptomycin resistant bacteria. It is also observed that the activity of these two derivatives was also equal to that of a broad- spectrum antibiotic gentamicin, however the activity was lower than that of ciprofloxin. The results of the present investigations suggest that 3b and 3c are important derivatives for further investigations for the therapeutic value in managing diseases caused by the test bacteria based on toxicological and pharmacological study.
